# Supplementary material for: Modeling glioblastoma heterogeneity as a dynamic network of cell states
Source: Mol Syst Biol. 2021 Sep 16;17(9):e10105. doi: 10.15252/msb.202010105 (PMC8444284; doi:10.15252/msb.202010105)
Supplement: Supplementary file 5 — Source Data for Figure 3 [file MSB-17-e10105-s001.zip › Figure3A_sourcedata/GSEA_3065/nosigdb_state5.GseaPreranked.1622718435914/ALLEN_114_OPCL1_6MYT1.html]

Details for gene set ALLEN\_114\_OPCL1\_6MYT1[GSEA]

|  || Dataset | state5 |
| Phenotype | NoPhenotypeAvailable |
| Upregulated in class | na\_pos |
| GeneSet | ALLEN\_114\_OPCL1\_6MYT1 |
| Enrichment Score (ES) | 0.8357559 |
| Normalized Enrichment Score (NES) | 2.2404037 |
| Nominal p-value | 0.0 |
| FDR q-value | 0.0 |
| FWER p-Value | 0.0 |
Table: GSEA Results Summary

  

Fig 1: Enrichment plot: ALLEN\_114\_OPCL1\_6MYT1      
 Profile of the Running ES Score & Positions of GeneSet Members on the Rank Ordered List

  

| PROBE | GENE SYMBOL | GENE\_TITLE | RANK IN GENE LIST | RANK METRIC SCORE | RUNNING ES | CORE ENRICHMENT || 1 | PTPRZ1 |  |  | 12 | 0.862 | 0.1399 | Yes |
| 2 | PTN |  |  | 19 | 0.824 | 0.2743 | Yes |
| 3 | SOX6 |  |  | 28 | 0.754 | 0.3970 | Yes |
| 4 | PLEKHH2 |  |  | 106 | 0.520 | 0.4736 | Yes |
| 5 | SEMA5A |  |  | 111 | 0.516 | 0.5577 | Yes |
| 6 | PDGFRA |  |  | 175 | 0.460 | 0.6260 | Yes |
| 7 | VCAN |  |  | 191 | 0.444 | 0.6971 | Yes |
| 8 | MAML2 |  |  | 220 | 0.425 | 0.7636 | Yes |
| 9 | MEGF11 |  |  | 702 | 0.261 | 0.7527 | Yes |
| 10 | DOCK10 |  |  | 751 | 0.250 | 0.7883 | Yes |
| 11 | OLIG1 |  |  | 809 | 0.239 | 0.8211 | Yes |
| 12 | NXPH1 |  |  | 990 | 0.212 | 0.8358 | Yes |
| 13 | NKAIN3 |  |  | 1766 | 0.129 | 0.7705 | No |
| 14 | NCKAP5 |  |  | 1790 | 0.127 | 0.7888 | No |
| 15 | COL11A1 |  |  | 2950 | 0.055 | 0.6686 | No |
| 16 | CA10 |  |  | 3556 | 0.025 | 0.6052 | No |
Table: GSEA details [plain text format]

  

Fig 2: ALLEN\_114\_OPCL1\_6MYT1: Random ES distribution      
 Gene set null distribution of ES for **ALLEN\_114\_OPCL1\_6MYT1**
